# “I Do Not Take My Medicine while Hiding” - A Longitudinal Qualitative Assessment of HIV Discordant Couples’ Beliefs in Discordance and ART as Prevention in Uganda

**DOI:** 10.1371/journal.pone.0169088

**Published:** 2017-01-12

**Authors:** Rachel King, Jiho Kim, Mastula Nanfuka, Murisho Shafic, Maureen Nyonyitono, Florence Galenda, David Moore

**Affiliations:** 1 Global Health Sciences, University of California San Francisco, San Francisco, California, United States of America; 2 BC Centre for Excellence in HIV/AIDS, Vancouver, British Columbia, Canada; 3 The AIDS Support Organization, Jinja, Uganda; 4 Faculty of Medicine, University of British Columbia, Vancouver, British Columbia, Canada; National and Kapodistrian University of Athens, GREECE

## Abstract

**Background:**

HIV negative members of serostatus discordant couples are at high risk for HIV acquisition, but few interventions are in place to target them in sub-Saharan Africa.

**Methods:**

In this study, we interviewed 28 couples, 3 times over a period of one year to understand their perceptions and attitudes around discordance, their relationship dynamics, their HIV risk behaviour, their beliefs and attitudes about antiretroviral therapy (ART) and their views of the community perceptions of discordance and treatment for HIV.

**Results:**

Findings revealed that at baseline there were multiple complex explanations and interpretations about discordance among discordant couples and their surrounding community. Shifts in beliefs and attitudes about discordance, HIV risk reduction and ART over time were enabled through re-testing negative members of discordant couples and repeat counselling but some beliefs remain solidly embedded in cultural imperatives of the importance of childbearing as well as culturally determined and enforced gender roles.

**Conclusions:**

Interventions that aim to target discordant couples must embrace the complex and dynamic understandings of HIV diagnosis and treatment in context of fluid relationships, and changing beliefs about HIV risk and treatment.

## Introduction

Expanded access to antiretroviral treatment (ART) has significantly reduced HIV-associated mortality and has likely contributed to reduced HIV incidence globally. An estimated 1.4 million new infections occurred in Sub-Saharan Africa in 2014, accounting for approximately 70% of new infections worldwide [1]. Heterosexual transmission among discordant couples in marriage or cohabitation accounts for most of the HIV incidence in this region [2] [3–6]. In sub-Saharan Africa, in studies where both members of the couple have been tested, rates of serodiscordance range between 1–7% [4, 5, 7–9] and 10–25% of new HIV infections occur among HIV-discordant couples [10]. In a study of five African countries using Demographic Health Survey (DHS) and AIDS Indicator Survey (AIS) data, de Walque found that among 9,297 infected persons, more than two thirds were in serodiscordant relationships [11].

More new HIV infections occur in stable discordant couples in sub-Saharan Africa than in any other group [12–14]. Coburn and colleagues designed a mathematical model to assess HIV transmission rates in discordant couples in 14 African countries and found that transmission can be as low as 1.9 per 100 person-years to as high as 19.0 per 100 person-years with the difference depending on the proportion of the population in stable relationships [15]. Studies that assessed risk for transmission among discordant couples found that in Uganda, factors such as lack of male circumcision, cohabitation, and low CD4 counts were associated with being seroconcordant HIV positive rather than serodiscordant [16]. In Kenya, additional factors included HSV2 infection in both partners [8]. The risk for women principally comes from the sexual behaviour of their sexual partner.

There have been both behavioural and biomedical research studies and programs focused on couples for HIV prevention. A systematic review conducted by Burton and colleagues found that couples-focused behavioural interventions reduced unprotected sexual intercourse and increased condom use compared with control groups [17]. Most of the couples-focused behavioral interventions in Africa are based on couple counseling and testing and have shown positive results including increased condom use from 4% to 57% in one year in Kigali Rwanda [8] and reported reduction of unprotected sex in Kenya, Tanzania and Trinidad [18]. Particular features of some of the successful interventions included a particular focus on gender dynamics and strengthening couple communication [19], counselor-facilitated disclosure [20] and increased number of follow-up visits after initial HIV diagnosis and counseling [21].

Recent biomedical prevention studies have focused on reducing HIV susceptibility and infectivity in sero-discordant couples meaning treating either the HIV-infected partner to reduce viral load using an approach called Treatment as Prevention (TasP) or treating the HIV-negative member of the couple to prevent acquisition called Pre-exposure prophylaxis (PrEP). Treating the infected partner with ART to prevent transmission to the uninfected partner found a prevention effect of 96% [22–24]. A recent systematic review and meta-analysis of per-partner HIV-1 infectiousness that included all published prospective studies of discordant couples, found an overall 91% reduction (95% CI = 79%-96%) in infectiousness with ART use by the index case from ART-stratified observational studies [2].

Previous studies have found beliefs in “stronger blood”[25], “spirits and supernatural forces” associated with HIV infection [26], HIV ‘hiding’ in the blood, or the belief that gentle sex will protect the sero-negative partner, [27] which may contribute to some challenges related to uptake of ART [28].

We conducted a longitudinal qualitative study to explore the perceptions of members of HIV serodiscordant couples over time in terms of their understanding of serodiscordance or eventual seroconversion and their sexual and adherence behaviour with regards to risk for seroconversion in the context of an observational cohort study of TasP in rural east-central Uganda. By interviewing individual couple members multiple times with the same interviewer, the aim was to build enough trust to limit social desirability bias that is inevitable with highly sensitive topics such as sexual behaviour particularly with a cross sectional design. This manuscript builds onto an analysis of the ‘baseline’ data.

## Methods

Jinja district, where this study was set, is in Eastern Uganda along the route to Kenya. It houses one of the most populated towns in Uganda with an estimated population of 90,000 and is about 80 km away from the capital, Kampala. Couples were clients of The AIDS Support Organization (TASO) in Jinja, Uganda. TASO is the oldest and largest non-governmental organization delivering HIV care and treatment in Uganda and provides treatment and support to over 100,000 HIV-affected clients through 11 service centers across the country. The ART program at TASO-Jinja was initiated in 2004. TASO has provided services for discordant couples such as support groups at all TASO centres as part of their routine programming. The 2014 WHO guidelines of recommending ART for HIV positive individuals whose regular sexual partners are HIV negative have been adapted by Ugandan Ministry of Health [29]. These guidelines had not yet been adapted in Uganda at the time of this study and it is not known how widely the understanding of TasP benefits have spread to HIV care and treatment programs staff and community.

The participants of this study were purposively selected from an observational study of TasP among sero-discordant couples known as the Highly Active Antiretroviral therapy as Prevention (HAARP) study. The HAARP study compared HIV incidence between HIV negative members of sero-discordant partners who were or were not receiving ART during the study and was conducted from June 2009 to December 2011. The study did not find a benefit from ART in terms of preventing HIV seroconversion in the negative participant [30]. To follow-up on these findings, we enrolled a selection of previous study participants in a qualitative sub-study for a series of three in-depth interviews beginning in June 2013 and continuing until August 2014.

A total of 28 couples, or 57 individuals were recruited into the study through a purposive selection process. Though almost all participants reported polygamy only, one “couple” enrolled both partners of the polygamous relationship (one husband and two wives). All couples were initially sero-discordant upon recruitment into the HAARP study. For this qualitative sub-study we purposively selected these individuals who had seroconverted. Their partners were also enrolled. We also recruited 14 age and gender-matched control HIV-positive participants whose partners did not seroconvert during the study period. The seropositive participant was asked to bring the seronegative partner into TASO to receive education and counselling about serodiscordance and prevention of transmission. By the beginning of the interviews, 14 of the couples had become sero-concordant.

Beginning in July 2013, trained interviewers carried out face-to-face individual in-depth interviews, with each member of the couple interviewed separately. Interviews were conducted at the TASO clinic site at Jinja, Uganda. The interviewers were male and female TASO caregivers, comprised of 1 physician, 2 nurses, and one counsellor. Participants were compensated with 20,000 Ugandan shillings (approximately $8 USD) at each interview for transportation costs and were not interviewed by their counsellor. All interviews were gender-matched between the participant and the interviewer. Interview guidelines included themes around the couples’ perceptions of sero-discordance, the community’s perception of serodiscordance, the couples’ sexual behaviour and relationship dynamics, desire to have children, opinions regarding HIV prevention strategies and the role of ARVs with respect to HIV prevention and treatment. For couples where seroconversion occurred, more in-depth discussion included their perceptions of why this occurred. The interviews were carried out in the language preferred by the participant (Luganda/Lusoga), audio recorded, then transcribed and translated into English. Nobody else was present during the interviews.

Analysts were trained on qualitative data analysis and specifically on coding our data using NVivo software. We used thematic coding and framework analysis as the primary analytic strategy. Framework analysis uses four analytic stages: familiarization (reading multiple times), identification of themes (developing the codebook), indexing and charting that involves arranging summaries of the data into a database according to theme, sub-theme, category and interpretation [31].

**Fig 1:**
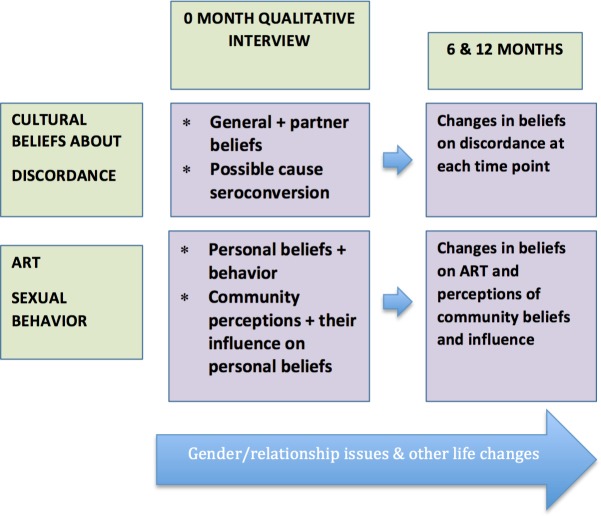
Methodology of the HAARP Longitudinal Qualitative Study and Domains Addressed.

Thus, after reading three transcripts, the analysis team members (comprised of five members) collaboratively developed a codebook of themes based on the interview topics as well as those emerging from the data. Three more transcripts were then reviewed to include additional topic areas and themes. This process was repeated until the codebook reached a stage where no new themes or topic areas emerged. All transcripts were then coded using the final version of the codebook before themes were summarized across respondents and over time. Analysis focused on identifying the dominant themes and the range of explanations for sexual behavior, motivations for preventive behaviour, treatment and comparisons across participants and over time. As shown in Fig 1, emphasis was placed on changing or emerging interrelationships from one time point to the next. [32]. Interviews attempted to build onto previous interaction with the interviewer, build trust and minimize social desirability. Interactive discussions were held with the analysis team to validate data interpretations and resolve any interpretation discrepancies. The final analysis framework looked like Table 1 below and was color-coded to distinguish visit times/ and differences between couples where a seroconversion took place and where it hadn’t. All non-converted couples were shaded in the table. The table was populated with both notes and quotes. Quotes were identified to best describe the point in the text and were described with attributes as follows: gender, age, number of children, HIV status, visit.

**Table 1:** Framework analysis of all HAARP Longitudinal Qualitative participants by theme and visit; N = 28 couples.

ID, participant /couple characteristics (age, HIV status, # children)	Relationship issues (eg. # women in ‘couple’, changes)	Condoms or other prevention	discordance thoughts/beliefs	Community discordance thoughts / beliefs	Community treatment beliefs	Seroconversion beliefs
Baseline—woman						
Baseline -man						
6 mos—woman						
6 mos–man						
12 mos–woman						
12 mos–man						

All interviews were conducted and recorded with the participants’ written consent. The study received approval from the Research Ethics Board of the University of British Columbia in Vancouver, Canada and the Science and Ethics Committee of the Uganda Virus Research Institute and the Uganda National Council for Science and Technology in Uganda.

## Results

### Study Participants

Of the 586 couples in the main HAARP study, 28 couples were enrolled in the qualitative sub-study. Of these 28 couples, 6 had the female member initially seropositive compared to the main study where 44% had a female seropositive at enrolment. At baseline,16 of the couples (57%) stated that they were involved in polygamous relationships; however over time almost all male couple members mentioned multiple partners at some point in their lives. The average participant was 42 years old and the median number of children in the main study was eight; in the sub-study the average (and median) number of children was 6. All clients consented to the qualitative interview process.

### Couples’ beliefs around discordance and changes over time

At initial qualitative interviews, all HIV positive participants had been TASO members for at least 3 years and over 40% had been TASO members for >5 years, thus having multiple HIV tests, many participants described feelings of confusion and disbelief when asked to discuss their HIV serostatus discordance. Almost all individuals claimed that when they received their test results, they did not believe them with multiple and varied explanations for their discordance beliefs. A frequent description of their beliefs was mistrust of the testing process. Both couple members often stated that the testing machines could have been failing, or that the virus was hiding. Another widespread explanation for discordance was the possibility of resistant blood.

Additionally, blood type was commonly mentioned together with the possibility of blood being able to hide the virus. Some participants who seroconverted believed that the virus had been hiding in their blood the whole time and that seroconversion was inescapable. Other explanations for discordance included god’s will or the belief that any health problem must indicate HIV is present. One HIV-negative woman was still sure she was positive because she would feel feverish, or “*these days I have developed serious chest pain and sometimes I even fail to move or stand up*. *Then sometimes*, *a part of my body gets paralyzed or* (I feel) *sharp pain and I end up crying in pain and I think that it is because of the virus* (Woman, 38 years, 9 children, HIV-negative, 6-months).

Some participants had no explanation at all for discordance, and expressed continued puzzlement.

The understanding that discordance was possible generally did not come quickly. Multiple negative test results and continuous counselling enabled participants to believe in their discordant status with time. Some participants changed their understanding and explanations over the course of the 12-month study. One participant described how at his first HIV test,

*“I felt powerless just like I used to feel back then when I was still in school before doing an exam*, *I think we used to call it examination fever*…*Laughing*…*you see even before I contracted the virus*, *she (my first wife) told me that I had to start using condoms when having sex with her as a means of preventing me from contracting the virus*… *At first I didn’t take her words seriously and she invited a counsellor to our home who talked to me and helped me understand so many things*… *After that talk with the counsellor I totally changed my mind on what I was going to do because I was planning to send away my wife so that I stay with my second wife since me we were not infected*… *After the two visits from the counsellor*. *I changed my attitude*. *That is why I came to a conclusion that I contracted the virus through the wrong use of the condoms*. (Man, 51 years, 11 children, converted, 12-months).

The participant above insisted that they were using condoms correctly at baseline interview.

An HIV-negative woman described how her beliefs in remaining negative changed over the course of many years. Remaining in good health and receiving multiple negative HIV test results with counseling enabled her to finally accept her HIV-negative results. When she and her husband first tested and received discordant results she said,

*I could not believe it*. *I always thought that we were both positive*. *We just kept on going with the results that we were getting from the tests*. *Now*, *yes*, *I do believe that I am negative because I do not test positive*. *Whenever I go for checkups*, *I am told that I am hypertensive and also at times that I have malaria and not AIDS*. (Woman, 40 years, 8 children, HIV-negative,12-months)

We describe in Table 2 below a summary of participant discordance explanations over time.

**Table 2:** Summary of discordance explanations among 28 couples at baseline, 6 months and 12 months interviews.

	Baseline	6-months	12-months
Believed negative or discordant or not sure of discordance	• *confused about negative result* • *believe negative so can take care of children when partner dies*	*Many years in discordant relationship; used to it*	• *Very healthy* • *Multiple negative tests* • *Many couples in similar situation*
Did not believe negative/discordant	• *Strong resistant blood* • *Blood type* • *Virus hiding* • *Machines faulty*	*Inconsistent condom use so not realistic to remain discordant*	*Small veins; virus hiding Blood group B which is weakest blood group*

### Wider cultural beliefs in discordance

At baseline, most couples said that community members did not believe in the possibility of discordance, often dismissing it outright. However, at later interviews, both 6-month and 12 month, participants reported having seen some changes in community attitudes or having learned how to cope with community disbelief. One woman who did not seroconvert over the project period explained that she continues to tell her neighbours that in fact discordance exists. She then stated:

*At first people used to think that being started on ARVs meant that one was going to die but they have started learning slowly by slowly that ARVs actually help*… *This is as a result of watching people that were badly off get better after taking ARVs to the extent that even when you meet them*, *you cannot know that they are HIV positive* (Woman, 40 years HIV-negative, 6 months).

Some participants explained at their 12-month interviews that they did not discuss having discordant results outside of their homes or TASO. One HIV-negative woman described her situation as:

*The people think that we are both positive*. *I do not tell people that I am negative and my husband is positive*. *Nobody knows*… *I have never told anybody*. *I tell them that we are both sick and on medication*. (Woman, 40 years, 8 children, HIV-negative, 12 months)

Overall the attitudes of community members regarding discordance were predominantly uninformed, and generally less tolerant than couple members, though views shifted slightly over time.

### Antiretroviral treatment; beliefs, fears, myths, and experiences in the community over time

The seropositive members of the couples were all grateful regarding the health benefits ART had provided them and their partners. Many mentioned the side effects upon ART initiation but few described side effects that lasted more than 6 months.

Participants were asked about what they believed community member’s views on ART were. These perceptions were quite mixed. At our first qualitative interview, many cited the belief that ART was given to individuals who were close to death, either to hasten or slow down their death. Some stated outright that they should stop taking ART as the drugs were harmful.

*Most people back then*, *before they got to know the importance of the medicine would say*, *“They brought this medicine to kill us*. *Most of them still think like that*… (Woman, 39 years, 6 children, Converted, 12-months).

However some noted that,

*ARVs weaken HIV and the speed at which it travels in the blood* (Woman, 47 years, Converted, 7 children, Baseline).

Over time, community opinions appeared to shift to greater understanding of the benefits of ART; many mentioned that they or their partner’s CD4 count changed; they or their partner became stronger and were able to work again,

*You end up getting rejuvenated and people start saying*, *“That man or that woman was on the verge of death but do you see what that medicine can do"* (Female, Converted, 47 years, 7 children, 6-months).

Some also correlated benefits of treatment such as long life, to the TASO program and how TASO provides more than strict biomedical care.

*So they say that if someone is in TASO*, *he or she can live longer than an HIV-negative person and even know how to plan* (Man, 52 years, 12 children, HIV positive, 12-months).

In addition, the 12-month interviews, though some participants still said that they have not disclosed discordance to their neighbours, raised the point of a slow shift in medicine-related stigma.

… *I do not take my medicine while hiding*. *If have gone to a funeral*, *it is my tin that first goes into the bag*. *If there is no water or if I am far from water*, *I say*, *“Please send me*…*” I call it ‘life’*. *“Please send me water so that I take ‘life’*.*” I then pick my tin and take*. *I do not take it while hiding* (Woman, 39 years, 6 children, Converted, 12-months).

The relationship between sexual behaviour and ART beliefs was complex. Some participants believed that taking ART increases risk of transmission and some believed that it decreased transmission. These beliefs did not seem to change over time or differ between men and women.

*“If you are on this medication […] if you have sex outside marriage you can get another kind of HIV […] when they mix*, *the one that you have wakes up*” (Woman, Converted, 27 years, 2 children, 12 months)

### HIV risk behaviour and sero-conversion over time

Participants were asked to describe their risk reduction behaviour both before and after seroconversion at each interview. At initial interview, there were many challenges noted with current (at that time) condom use. Some men mentioned that that were not confident with how to use condoms, that their wives complained of pain when their partners used condoms, some disagreed on their use, some mentioned that they actually did not understand the importance of condom use if they believed they were HIV-positive and many acknowledged that they did not use condoms consistently.

*We knew or believed that we were both infected and so did see any importance of using* [condoms]” (Man, 52 years,12 children, HIV-positive, Baseline).

At the 6-month visit, participants were consistent in their inconsistent condom use. Couples often mentioned that they changed their condom use behaviour after receiving sero-conversion results. Explanations for consistent and inconsistent use included, protecting oneself, both for those who are sero-negative and those who are seropositive.

One woman at her 6-month visit mentioned that, *We protect ourselves from them* (from condoms) *by reducing the number of times we have sex and still when we have sex*, *we use condoms* (Woman, 32 years, 5 children, converted 6-months).

For seropositive respondents, a common reason they cited is a need for protection from increased amount of HIV, another ‘type’ of HIV or from other STIs.

*We use condoms; not every time it helps me not to add on to the virus that I have* (Woman, Converted, 47 years, 7 children 6 month visit)

Other participants asserted that their husbands refused to use condoms consistently or that their wives complained of pain caused by condom use.

By the 12 month interviews, risk-reduction behaviour overall appeared to be more stable. There were a few participants who remained inconsistent condom users, but many had shifted to reporting that they were using condoms every time they had sex. This behaviour was often combined with decreasing frequency of sex.

*At times we use a condom though sometimes my husband doesn’t want to use condoms but when that happens we don’t have sex because I cannot accept to have sex without a condom* (Woman, 40 years, 6 children, Converted, 12-months).

Men and women were generally reporting similar behaviour and the motivation for condom use was sometimes in relation to the shock of seroconversion results.

*In my life*, *I cannot have sex with a man without using a condom*. *I knew that I needed my life but all I was left with were regrets*. *By the time you decide to act*, *it is normally too late*… *After getting such a problem as HIV*, *you get used to them and start liking them* (Woman, converted, 47 years, 7 children, 12-months).

Often couples who reported unwavering condom use described having normalized the behavior.

*Nowadays it even feels like I don’t have a condom on because am used to them* (Man, 49 years 9 children, converted 12-months).

As shown in the summary Table 3 below, participants cited biomedical, behavioural, relational and cultural explanations for their or their partners’ seroconversion over the study period. Sometimes the reason was unprotected sex, but the more important factor may have been a participant’s desire or partner’s desire for a child or male child. Reasons related to partners or relationships and child-bearing were consistently mentioned multiple times over all time points for both men and women. The shifts that did occur over time could have been related to greater trust in the interviewer, a belief in the effectiveness of condom use or that one’s own behaviour must have caused the seroconversion.

**Table 3:** Summary of Participant Explanations for Seroconversion over time.

	Baseline	6-months	12-months
Cultural/religious	*- must be God’s will*	• *Need for baby or baby boy* • *maybe the virus was hidden the whole time*	*- the virus was hidden the whole time*
Relational	*- co-wife became pregnant*	*Husband’s other women/girlfriends*	*-husband wants more children*
Behavioral/biomedical	• *wounds in genitals* • *unprotected sex*	*-gaps in condom use*	• *not using condoms* • *my own behavior (many women)*

**Fig 2:**
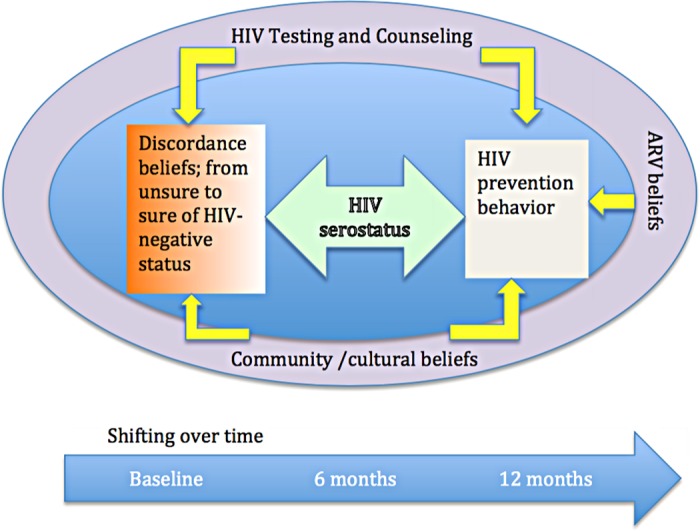
Conceptual Framework of Emerging Themes over Time.

As shown in the Fig 2 below, beliefs ranged from sure of negative serostatus, to unsure and these beliefs were related to ones experience with HIV testing and counselling, ones serostatus and the community beliefs surrounding discordance. HIV prevention behaviour, reduction in frequency of sex, condom use and changes in partners were also related to HIV serostatus, discordance beliefs, ARV beliefs and community understanding of discordance.

## Discussion

Our main findings of gender-matched interviews with both members of serodiscordant couples over a 12 month period in rural Ugandan revealed complex beliefs and behaviours in relation to discordant HIV serostatus, risk reduction, couples relationships in relation to gender roles and fertility, and anti-retroviral therapy and that these beliefs and behaviours did shift over time or participants were more open about reporting. A majority of participants had limited biomedical knowledge of serodiscordance at the first interviews, despite having previously been participants in an observational study of serodiscordant couples where couple counseling was provided every 6 months. Couples showed culturally sound reasoning for their lived experiences of being part of a discordant relationship. Some of the reasons given, such as the existence of strong blood and the virus in hiding, have been reported in other studies of HIV sero-discordance in Africa. [25, 27, 33] These beliefs may influence prevention behaviour. [34] As shown in Fig 2, there are multiple factors that influenced these couples’ beliefs and behaviour over time. Couple members’ past or current sexual behaviour, condom use, multiple relationships influenced whether or not they believed their discordant status. Many couples cited their previous or current unprotected sex as a reason not to believe that they were discordant. In the same vein, unprotected sex was a reason to have understood why some participants seroconverted.

Community knowledge of ART has changed slightly over time, but negative beliefs were still noted in our study. Other studies found similar ART beliefs despite having been conducted nearly 10 years before the current study.[27]

Several couples reported consistent condom use after finding out their serodiscordant status and also often changed over the course of the 12-month study. This change in sexual behaviour after testing has been reported in many sub-Saharan studies, especially when accompanied by counselling and education but was not reported in our observational HAARP study. [35] [13, 26, 30]

Multiple factors were at play in the lives of these couples over the period of this study, including the cultural imperative to have children, especially male children. This has been highlighted in many studies based in Uganda and the region and often overrides other factors such as the risk of HIV acquisition.[9, 35, 36]

As this study was designed to explore couple dynamics over time, the unit of analysis was the couple and thus we attempted to take into consideration the dyadic aspects in each of the categories we were looking at. In addition we wanted analyze changes over time; attempting to see emerging interrelationships between categories. [32] Thus, the complexity was layered by the dynamic context, actors and issues we were assessing. The importance of couple interactions and communication in relation to health behaviour in the literature highlights how the complex dynamics in these relationships have a powerful impact on couples’ ability to adhere to treatment. [35]. In South Africa, Conroy also studied the effects of power and relationship dynamics with regards to health behaviour and found that to improve HIV-related behaviors it is critical to consider sources of shared power within couples and think about empowerment at the couple level. She suggests that efforts should be made to take the dyadic environment into consideration as well as men's perspectives to ensure positive relationship and health outcomes. [37] We found that the ‘couple’ in our setting, was often a polygamous one, and thus, sometimes one couple member would cite a third person as the potential source of infection. Often, a wife would mention that she knew the HIV-status of her co-wife, but in other couples, a participant would simply say she didn’t know the risks her husband was taking when not at home. We also noted that couple dynamics often changed over the different time points.

Participants reported at baseline that the wider community did not believe that discordance was possible. What appeared to change over time with respect to community beliefs was participants’ reaction to these community beliefs. Once participants believed that they were discordant, community perceptions seemed to matter less.

## Limitations

Our participants, as TASO clients, were previously HIV-tested, counselled and followed-up in a cohort study. Thus, they likely do not represent serodiscordant heterosexual couples in rural African settings who are not enrolled into similar programs. However, given that these individuals appear to have varied beliefs and perceptions about HIV serodiscordance that are not commonly held by the health care personnel, it is likely that these views are held by others. Although efforts were made to work with interviewers on rapport building and thus eliciting answers in a comfortable, trusting environment, some social desirability bias may exist.

In the current test and treat environment in sub-Saharan Africa where there is increasing evidence of significant reductions in transmission within couples on ART, public health imperatives are putting more people on treatment to decrease community viral load. This highlights the importance to understand how couples perceive their dyadic serostatus and their need or no need for treatment and prevention behaviour in order for successful rollout of TasP.

## Supporting Information

S1 File (DOC)

S2 File (DOC)

S3 File (DOCX)

S4 File (DOC)
