# Supplementary material for: “I Do Not Take My Medicine while Hiding” - A Longitudinal Qualitative Assessment of HIV Discordant Couples’ Beliefs in Discordance and ART as Prevention in Uganda
Source: PLoS One. 2017 Jan 12;12(1):e0169088. doi: 10.1371/journal.pone.0169088 (PMC5232346; doi:10.1371/journal.pone.0169088)
Supplement: S1 File — (DOC) [file pone.0169088.s001.doc]

**Interview Guide for HIV Negative Partners**

**Follow-up visit -1 (3 months)**

| **SECTION** | **1** | **INTERVIEW PARAMETERS** |
| --- | --- | --- |

|  |  | Start time of interview |  |
| --- | --- | --- | --- |
|  |  | Date of interview |  |
|  |  | Serostatus (positive, negative, seroconverter) |  |
|  |  | Gender |  |
|  |  | Survey respondent ID number |  |
|  |  | Test phrase |  |
|  |  | Interviewer name |  |
|  |  | End time of interview |  |
|  |  | Next appointment date |  |

| **SECTION** | **2** | **INTRODUCTION** |
| --- | --- | --- |

As we discussed in your last visit 3months ago, you have been asked to participate in this study on discordant couples. I know that this topic of discordance has been confusing in the past. So please feel free with me and let’s discuss your experiences and situation. If you agree, I would like to take some notes during our conversation and also tape record our discussion so we don’t lose any important information.

** Note: Please adapt the guide to the specific situation (man/woman)*

| **SECTION** | **3** | **RESPONDENT CHARACTERISTICS**  **Focus on what has changed since last visit** |
| --- | --- | --- |

I would like to learn more about you. How old are you now?

How many children do you have?

Has this changed since last visit?

Where do you stay? Have you changed residence since your last visit?

Are you still in a relationship with the partner from the HAARP study? Are you in a polygamous relationship (do you/does your husband have more than one wife)?

| **SECTION 4** | **UNDERSTANDING DISCORDANCE AND TRANSMISSION RISK** |
| --- | --- |

**Sub-topic 1: How does the HIV negative person understand their sero-status?**

Let’s talk about your HIV status. Tell me what you think about your HIV status. What to add since last visit?

PROMPT:

1.1 Have you tested for HIV since your last visit?

1.2 Why do you think you received an HIV negative result? Any new thoughts?

1.3 What do other people think about discordance? Do other people’s beliefs influence your own beliefs about whether you are truly discordant? In what ways? Tell me more about your **thoughts that may be different from last visit.**

**Sub-topic 2: How does the HIV positive person understand their risk of transmission?**

2.1 How would you feel if you got an HIV positive result? Tell me more about that. (***PROBE)***

2.2 Do you think your current sexual behaviors put you at risk of acquiring HIV from your spouse/ partner? Why? Why not? Tell me more… What has changed since last visit? Do you have the same partner as the last visit?

***If relevant*** - How does your current sexual behavior differ with different sexual partners?

2.3 ***If relevant*** - Do you think there is anything special in your body that makes you HIV negative? Tell me more about that…

**Sub-topic 3: Risk reduction strategies**

3.1 What are the things you and your partner(s) have done to reduce your risk of getting HIV through sex? Tell me more about this… *(Probe, eg if couple is using condoms, how frequently; frequency of sex* ***and changes since last visit****)*. Please describe when have you found it difficult to do these things? ***If relevant*** - Is it different with different partners?

3.2 What have you done as an individual to reduce the risk of transmission ? Tell me more about that. Please describe how it has been different with different partners?

3.3 How about your partner(s) - tell me what your partner(s) has/have done to help reduce your risk of getting HIV.

3.4 What has worked well? Why?

3.5 What difficulties have you experienced in trying to reduce your risk of getting HIV infection? Anything else? *(Probe and prompt as needed for specific issues like attitudes towards condom use, lack/poor couple communication on sexual issues, attitudes within the couple about each other, personal beliefs and attitudes about discordance, norms about sexual satisfaction-e.g. fatalism (already infected, helplessness over the control of HIV positive partner, etc)*.

3.6 Tell me about your experiences using condoms with your partner; especially since last visit.

a. *If a couple is NOT using condoms, or not using them consistently*:

Why have you decided not to use condoms? What prevents you from using condoms? Any other reasons/problems?

***If relevant -*** Please explain if it is different with different partners.

b. *(If couple is using condoms, ask about condom availability and use)*

Tell me about your experience using condoms with your partner.

How do you get your condom supply?

Do you ever have difficulty getting sufficient condoms?

Do you and your partner(s) ever have disagreements about condom use? Tell me more.

Tell me about any other problems about using condoms? (***Ask for individual, partner-specific, structural barriers)***

What strategies have you adopted to help you and your partner(s) use condoms regularly and well?

***Prompt****, (if respondent has not mentioned any of the following risk reduction measures):*

3.7 “You have not mentioned abstinence, let’s talk about it”. Do you and any of your partner(s) abstain from sex? Why or why not?

3.8 Sometimes people having trouble abstaining completely but they are able to reduce the frequency of having sex. Tell me about how you think reducing the frequency of sex works for you and your partner.

3.9 Does alcohol use also play a role in whether or not you are able to reduce your risk?

How? /tell me more…

**Sub-topic 4 (*only for participants on ARVs*): Impact of ARVs on HIV risk for discordant couple**

4.1 How do you feel about your partner being on ARVs? How do you think being on ARVs affects your sexual life? Probe for positive and negative outcomes. Tell me more about this.

4.2 Do you think the ARVs increase the chance that you will acquire HIV? Why?

Do you think the ARVs decrease the chance that you will acquire HIV? Why? ***Probe on ARV effects on prevention behavior.***

4.3 **Since your last visit**; what other beliefs have people expressed about ARVs? How does this affect your sexual life? What do other people say you should do to reduce the risk?

- 1. Adherence to HAART *Find out if the partner is on HAART: If so, proceed with the probes.*

**It is often hard to take medicine every day and anyone could find it difficult.  We would like to know how your partner has managed HIV treatment doses within the last 30 days?**

*Find out what type of ART partner is taking. Ask them to describe how they are taking them*

**You can probe on challenges and coping mechanisms such as: side effects, getting refills, food insecurity, stigma, medicine companion (who reminds the participant to take medicine?), any use of other reminders, any pressure to share drugs?**

**What might be additional challenges, for those who missed some doses the reasons as to why they missed or how has the participant really managed so well (whichever might be relevant for the particular participant).**

**SECTION 5 SOCIAL NETWORKS**

Let’s talk about the kind of people you interact with or you meet on a daily basis. These may be friends, relatives, workmates, lovers or any other person. Please feel free to tell us about them.

5.1 What kind of people do you interact with since your last visit?

(PROBE: neighbors, relatives, family members, friends, workmates, health workers, girlfriends, boyfriends, spouse, students ?

5.2 What kind of personal support systems do you have? How has this changed since last visit? (*PROBE for emotional, financial, material, moral and psychological, housing, accessing health care, support through health providers, and assistance in violent situations*). Under what circumstances are your personal support systems formed? How are they formed? *(PROBE: word of mouth, mobile phones, etc)*

**SECTION 6 PARTNER RELATIONSHIPS**

6.1 Tell me about how things have been with your partner(s) over the past 3 months? How has HIV affected your relationship(s)?

6.2 How has your sex life been? (*if this hasn’t yet been covered)*

Prompts:

Do you feel satisfied sexually? Have you observed any changes in your sex life since last visit?

6.3 Do **you** desire for more children? If **yes** or **no** – PROMPT - Tell me more about this.

Does **your partner(s)** desire more children? Tell me more about this.

6.4 How committed are you to staying with your main partner? What about any other partners? What brings the attachment/commitment? How does this differ between partners? Tell me more about each of those partners (if relevant) [Possible prompts; when do you meet? Where do you meet? How often? How did you meet?]

6.5 Tell me about the communication with your partner(s) since last visit. What issues are easy and what issues are difficult to communicate about? (possible probes: condom use, FP, school fees, other partners, alcohol use)

6.6 Who makes the decision on using condoms? How are the decisions made? How does this differ between different partners?

**SECTION 7 Motivations for participant remaining HIV negative**

7.1 Tell me how you feel about being HIV negative. What are the benefits to you of remaining HIV negative? What are the benefits to your partner(s)? To your children (if any)? To your family? To the community?

7.2 Would there be any benefit to you becoming HIV positive?

7.3 Do you feel that your partner is concerned about giving HIV to you? Why or why not? If concerned, what are the challenges?

7.4 Other questions/comments/suggestions

Follow-up interview can discuss when relevant:

**How many sex partners** have you had sex with in the last 3 months?

Please describe how you relate with each one of them.

- *Partner type (steady, casual, regular, commercial, spouse)*
- *Individual characteristics of partner (age, ethnicity)*
- *Relationship with each partner (duration and nature of relationship, where/ when/ how met partner; if commercial)*
- *Probe around past sexual experiences with this partner*

**How is having sex with a “steady partner” different from having sex with others,** such as casual partners, one-night stands, commercial partners, etc
