# Supplementary material for: “I Do Not Take My Medicine while Hiding” - A Longitudinal Qualitative Assessment of HIV Discordant Couples’ Beliefs in Discordance and ART as Prevention in Uganda
Source: PLoS One. 2017 Jan 12;12(1):e0169088. doi: 10.1371/journal.pone.0169088 (PMC5232346; doi:10.1371/journal.pone.0169088)
Supplement: S3 File — (DOCX) [file pone.0169088.s003.docx]

**Interview Guide for the Index Partner of the Participant who Seroconverted during the HAARP Study**

**Follow-up visit -1 (3 months)**

| **SECTION** | **1** | **INTERVIEW PARAMETERS** |
| --- | --- | --- |

|  |  | Start time of interview |  |
| --- | --- | --- | --- |
|  |  | Date of interview |  |
|  |  | Serostatus (positive, negative, seroconverter) |  |
|  |  | Gender |  |
|  |  | Survey respondent ID number |  |
|  |  | Test phrase |  |
|  |  | Interviewer name |  |
|  |  | End time of interview |  |
|  |  | Next appointment date |  |

| **SECTION** | **2** | **INTRODUCTION** |
| --- | --- | --- |

As we discussed in your last visit 3months ago, you have been asked to participate in this study because you had a partner who became HIV positive during the TASO couples study. I know that this topic of discordance has been confusing in the past. So please feel free with me and let’s discuss your experiences and situation. If you agree, I would like to take some notes during our conversation and also tape record our discussion so we don’t lose any important information.

** Note: Please adapt the guide to the specific situation (man/woman)*

| **SECTION** | **3** | **RESPONDENT CHARACTERISTICS**  **For follow-up – focus on what has changed since last visit** |
| --- | --- | --- |

I would like to learn more about you. How old are you now?

How many children do you have? Has this changed since last visit?

Where do you stay? Have you changed residence since your last visit?

Are you still in a relationship with the partner from the HAARP study?

Are you in a polygamous relationship (do you/does your husband have more than one wife)?

| **SECTION 4** | **UNDERSTANDING DISCORDANCE AND TRANSMISSION RISK** |
| --- | --- |

**Sub-topic 1: How did the HIV positive person understand their sero-status?**

1.0 In the HAARP study, your partner tested positive for HIV. Why do you think your partner received an HIV positive result? Do you have any thoughts you would like to add to what you told us the last time?

1.1 Think back to before your partner tested positive for HIV: What did other people think about discordance? Did other people’s beliefs influence your own beliefs about whether you were a truly discordant couple? In what ways? Tell me more about that. Do you know other couples who have discordant HIV results that you’d like to talk about?

1.2 How did you feel when you found out your partner received an HIV positive result? Tell me more about that. (***PROBE) Are there additional thoughts you’d like to add to what you shared the last visit?***

**Sub-topic 2: How did the HIV positive person understand their risk of transmission**

2.0 Think back to before your partner tested positive for HIV: How did you feel about the possibility of your partner getting infected with HIV? Anything to add?

2.1 Do you think your sexual behaviors **currently** put others at risk of HIV transmission (if in a polygamous relationship)? Why? Why not? Tell me more…

***If relevant*** - How does your sexual behavior differ with different sexual partners; probe on condom use

**Sub-topic 3: Risk reduction strategies**

3.0 Think back to before your partner tested positive for HIV: What were the things you and your partner(s) did to reduce their risk of getting HIV through sex? Tell me more about this… *(Probe, eg if couple was using condoms, how frequently; frequency of sex etc)*. Please describe when you found it difficult to do these things? **Is there anything you’d like to add on from last time. Please describe any risky behavior you might have currently.**

***If relevant*** - Was it different with different partners?

3.1 If you had any other sexual partners near the time that your partner became HIV positive: What are the things you and your partner(s) did to reduce their risk of getting HIV through sex? Tell me more about this… *(Probe, eg if couple was using condoms, how frequently; frequency of sex etc.)*. Please describe when have you found it difficult to do these things?

***If relevant*** - Was it different with different partners?

3.2 What have you done as an individual to reduce the risk of transmission to your other current partner(s) in the present? Tell me more about that. What has worked well? Why? Please describe how it has been different with different partners?

3.3 Think back to before your partner tested positive for HIV: Tell me about your experiences using condoms with your partner. Please describe anything you did not tell us the last time.

a. *If a couple was NOT using condoms, or not using them consistently*:

Why did you decide not to use condoms? What prevented you from using condoms? Any other reasons?

b. *(If couple did use condoms consistently, ask about condom availability and use)*

Tell me about your experience using condoms with your partner before they became HIV positive.

How did you get your condom supply?

Did you ever have difficulty getting sufficient condoms?

Did you and your partner(s) ever have disagreements about condom use? Tell me more about that…, etc…

Tell me about any other problems about using condoms? (***Ask for individual, partner-specific, structural barriers)***

What strategies did you adopt to help you and your partner use condoms regularly and well?

3.4 Did alcohol use also play a role in whether or not you were able to reduce the risk of giving HIV to your partner? Anything to add?

How? /tell me more…

**Sub-topic 4 (*only for participants on ARVs*): Impact of ARVs on HIV risk for previously discordant couple**

4.0 a. *Think back to before your partner tested positive for HIV:*

How did you feel about being on ARVs?

How did you find being on ARVs affected your sexual life? Probe for positive and negative outcomes. Tell me more about this. **Anything to add?**

4.1 What are other peoples’ beliefs about ARVs? How does this affect your sexual life? How does ARV use affect your current sexual behavior?

4.2 Please tell me what you know about ARV drug resistance? Is this different from what you knew before you learned your partner was HIV-positive?

**4.3 Adherence to HAART by participant/partner:**

It is often hard to take medicine every day and anyone could find it difficult.

Tell us about your and your partner‘s ART adherence behaviour and how you would rate it?

You can probe on challenges and coping mechanisms such as: side effects, getting refills, food insecurity, stigma, medicine companion (who reminds the Index participant to take medicine?), any use of other reminders, any pressure to share drugs? Any changes since last visit?

4.4 Please tell me what you know about ARV drug resistance? Tell me more about that…..

4.5 Has your desire for children changed since you/your partner started taking ARVs? How? Tell me more about that… Any additional thoughts since last visit?

**SECTION 5 SOCIAL NETWORKS**

Let’s talk about the kind of people you interact with or you meet on a daily basis. These may be friends, relatives, workmates, lovers or any other person. Please feel free to tell us about them.

5.1 What kind of people do you interact with?

(PROBE: neighbors, relatives, family members, friends, workmates, health workers, girlfriends, boyfriends, spouse, students? Please tell me if any of the following have changed since last visit.

5.2 Which of these people have you told that you have HIV? How did they react?

5.3 What kind of personal support systems do you have? (PROBE for emotional, financial, material, moral and psychological, housing, accessing health care, support through health providers, and assistance in violent situations). Under what circumstances are your personal support systems formed? How are they formed? *(PROBE: word of mouth, mobile phones, etc.)*

**SECTION 6 PARTNERRELATIONSHIPS:**

6.1 Tell me about how things have been with your partner(s) over the past 3 months? How has HIV affected your relationship(s)?

6.2 Over the past 3 months, how has your sex life been? (*if this hasn’t yet been covered)*

Prompts:

Do you feel satisfied sexually? Have you observed any changes in your sex life, since you both learned that your partner is HIV positive?

6.3 Currently do **you** want more children? If **yes** or **no** – PROMPT - Tell me more about this.

Does **your partner** want more children? Tell me more about this.--What about your other sexual partners?

6.4 a. *Think back to before your partner tested positive for HIV:*

How committed were you to staying with your main partner? How many partners did you have? Please tell me about these partners. (PROMPTS: When did you meet? How frequently? Where?)

b. *What about since learning that your participant is HIV-positive?:*

How committed are you to staying with your main partner? What about any other partners? What brings about the attachment/commitment? How does this differ between partners?

6.5 Tell me about the communication with your partner(s). What issues are easy and what issues are difficult to communicate about? (Possible probes: condom use, FP, school fees, other partners, alcohol use)

*Are there any differences compared to before your partner tested positive for HIV?*

6.6 Who makes the decision on using condoms? How are the decisions made? What about before your partner tested positive for HIV? How does this differ between different partners?

**SECTION 7 Motivations for partner remaining HIV negative**

**Only if other partners:**

7.1 Tell me how you feel about your other partner(s) being HIV negative. What are the benefits to you if she/he remains HIV negative? What are the benefits to your partner(s)? To your children (if any)? To your family? To the community?

7.2 Would there be any benefit to she/he becoming HIV positive?

7.3 a) *Think back to before your partner tested positive for HIV:*

Did you feel that your partner was concerned about getting HIV from you? Why or why not? If concerned, what were the challenges he/she is facing?

b) What about in the present?

Do you feel that your other partners are concerned about getting HIV from you? Why or why not? If concerned, what are the challenges they are facing?

7.4 Other questions/comments/suggestions

Follow-up interview can discuss when relevant:

**How many sex partners** have you had sex with in the last 3 months?

Please describe how you relate with each one of them.

- *Partner type (steady, casual, regular, commercial, spouse)*
- *Individual characteristics of partner (age, gender, ethnicity)*
- *Relationship with each partner (duration and nature of relationship, where/ when/ how met partner; if commercial)*
- *Probe around past sexual experiences with this partner*

**How is having sex with a “steady partner” different from having sex with others,** such as casual partners, one-night stands, commercial partners, etc
