# Supplementary material for: “I Do Not Take My Medicine while Hiding” - A Longitudinal Qualitative Assessment of HIV Discordant Couples’ Beliefs in Discordance and ART as Prevention in Uganda
Source: PLoS One. 2017 Jan 12;12(1):e0169088. doi: 10.1371/journal.pone.0169088 (PMC5232346; doi:10.1371/journal.pone.0169088)
Supplement: S4 File — (DOC) [file pone.0169088.s004.doc]

**Interview Guide for participant who has seroconverted**

**Follow-up 1 (3 months)**

| **SECTION** | **1** | **INTERVIEW PARAMETERS** |
| --- | --- | --- |

|  |  | Start time of interview |  |
| --- | --- | --- | --- |
|  |  | Date of interview |  |
|  |  | Serostatus (positive, negative, seroconverter) just |  |
|  |  | Gender |  |
|  |  | Survey respondent HAARP ID number |  |
|  |  | Test phrase |  |
|  |  | Interviewer name |  |
|  |  | End time of interview |  |
|  |  | Next appointment date |  |

| **SECTION** | **2** | **INTRODUCTION** |
| --- | --- | --- |

As we discussed in your last visit 3months ago, you have been asked to participate in this study because you became HIV positive during the TASO couples study. I know that this topic of discordance has been confusing in the past. So please feel free with me and let’s discuss your experiences and situation. If you agree, I would like to take some notes during our conversation and also tape record our discussion so we don’t lose any important information.

** Note: Please adapt the guide to the specific situation (man/woman)*

| | **SECTION** | **3** | **RESPONDENT CHARACTERISTICS**  **For follow-up – focus on what has changed since last visit** | | --- | --- | --- | | **3** | | **RESPONDENT CHARACTERISTICS**  **Focus on what has changed since last visit** | | --- | |
| --- | --- | --- | --- | --- | --- | --- |

I would like to learn more about you. How old are you now?

How many children do you have?

Has this changed since last visit?

Where do you stay? Have you changed residence since your last visit?

Are you still in a relationship with the partner from the HAARP study? Are you in a polygamous relationship (do you/does your husband have more than one wife)?

| **SECTION 4** | **UNDERSTANDING DISCORDANCE AND TRANSMISSION RISK** |
| --- | --- |

**Sub-topic 1: How did the seroconverting participant understand their sero-status**

1.1 Think back to when you learned that you were HIV-positive. How did you feel about the HIV positive result? Tell me more about that . . . how has this changed since your last visit? Tell me more about your feelings and the circumstances around receiving the results.

1.2 Now that you have received a positive HIV result, which behaviors did you feel put you at risk of infection? Anything to add since last visit?

*Probe in detail in relation to what was discussed in above* ***e.g****., if respondent mentioned that they thought they had special blood, probe more about their opinion about transmission risk*

Since your last visit, do you feel your sexual behaviors put your partners at risk of getting HIV from you? Why? Why not? Tell me more…

**Sub-topic 2: Risk reduction strategies**

2.1 What are the things you and your partner(s) do to reduce your risk of getting STIs? Tell me more about this… (Probe, eg if couple is using condoms, how frequently; frequency of sex, etc).

When did you find it **difficult** to do these things?

2.1.1 How were you able to **discuss sexual issues** with your partner(s)? What worked for you in reaching this understanding? Tell me more. What difficulties did you experience in reaching an understanding with your partner(s)? Anything new since last visit?

2.2 What do you think worked well? Why?

If relevant - What strategies did you adopt to help you and your partner use condoms regularly and well?

2.3 What difficulties did you experience in trying to reduce your risk of infection? Anything else? (Probe as needed for specific issues like **attitudes towards condom use**, lack/poor couple **communication** on sexual issues, attitudes within the couple, personal beliefs and attitudes about discordance, **norms** about sexual satisfaction, witchcraft, fatalism (already infected, helplessness over the control of HIV positive partner, etc):

***Prompt (if respondent has not mentioned any of the following risk reduction measures): Prompt on updates from the last visit re questions in the following section.***

2.4 “You have not mentioned abstinence, let’s talk about it. Did you consider using this method as a means of avoiding HIV transmission in your relationship? Tell me more about that…Do you think you and your partner could abstain from sex? Why / why not?

2.5 Sometimes people have trouble abstaining completely but they are able to reduce the frequency of having sex. Did you consider using this method? Why? Why not? Tell me more about that…

2.6 Do you take alcohol? Does your partner take alcohol? Did alcohol use also play a role in whether or not you were able to reduce your risk? How? /tell me more…

2.7 What do you think would have made prevention easier for you and your partner?

**Sub-topic 3: Impact of ARVs on HIV risk for discordant couple (NOTE – only for couples where one or both partners are on ART). Please ask questions as updates from last visit.**

3.1 Are either you or your partner on ARVs?

How did you feel about you and/ or your partner being on ARVs?

How does being on ARVs affect your sexual life?

*Probe for positive and negative*. Tell me more about this.

3.2 Did you think that the ARVs increased/decreased the chance that you would get HIV? Why? Any additional thoughts since last visit?

How did the fact that your partner was on ARVs affect your use of risk reduction strategies/practices? Please explain. Any additional thoughts since last visit?

3.3 What are other peoples’ beliefs about ARVs? *Probe for interest in sex with current partner and outside partners.*

**3.4 Adherence to HAART by participant/partner:**

It is often hard to take medicine every day and anyone could find it difficult.

Tell us about you/ your partners ART adherence behaviour and how you would rate it?

You can probe on challenges and coping mechanisms such as: side effects, getting refills, food insecurity, stigma, medicine companion, any use of other reminders, any pressure to share drugs? Any changes since last visit?

3.5 Please tell me what you know about ARV drug resistance?

**SECTION 5 SOCIAL NETWORKS**

Let’s talk about the kind of people you interact with or you meet on a daily basis. These may be friends, relatives, workmates, lovers or any other person. Please feel free to tell us about them. Any additional thoughts since last visit?

5.1 What kind of people do you interact with? (PROBE: neighbors, relatives, family members, friends, workmates, health workers, girlfriends, boyfriends, spouse, students? Any additional thoughts since last visit?

5.2 What kind of personal support systems do you have? (*PROBE for emotional, financial, material, moral and psychological, housing, accessing health care, support through health providers, and assistance in violent situations*). Under what circumstances are your personal support systems formed? How are they formed? *(PROBE: word of mouth, mobile phones, etc)*

5.3 Among those people you mentioned, did you tell any of them that you were in a discordant relationship? How did they react? Any additional thoughts since last visit?

5.4 Among those people you mentioned, did you tell any of them that you became HIV positive? How did they react? Any additional thoughts since last visit?

**SECTION 6 PARTNER RELATIONSHIPS:**

6.1 Tell me about how things have been with your partner(s) since your last visit?

- Did you experience verbal abuse (insults, blame) from your partner since last visit? Tell me more about that…
- Did you experience any physical abuse (beatings, kicks, slaps etc) by your partner? Tell me more about that…

6.2 Since your last visit how was your sexual life with your partner(s)? ***Please again clarify *which* partner is being discussed.***

PROBE for positive and negative changes:

- Did you feel satisfied sexually?
- Have you had other relationships apart from the partner in the study? Please tell me more about them.
- Did you have unprotected sex against your will with your partner? Tell me more about that….
- Did you have unprotected sex with other sexual partners apart from your spouse? Tell me more about that…. What were the circumstances surrounding this/these relationship(s)? What is the HIV status of this/these partner(s)?

6.3 Do you (or your partner) desire for more children? If yes or no – PROMPT - Tell me more about this.

- Has your desire for children changed since last visit?
- Do you desire to prevent pregnancy? Why? Why not?
- If relevant - What are you currently doing to prevent pregnancy? What method are you using? How long have you used this method? Why did you choose this method? Tell me more about that. Are you having any difficulty with current method? Explain if so.
- What do you hope to do in future to prevent pregnancy? Why? Explain.

6.4 Other questions/comments/suggestions

Follow-up interview can discuss when relevant:

**How many sex partners** have you had sex with in the last 3 months?

**If you mention only one? Is this the one from HAARP? Please probe. If more than 1, please probe.**

Please describe how you relate with each one of them.

- *Partner type (steady, casual, regular, commercial, spouse)*
- *Individual characteristics of partner (age, work)*
- *Relationship with each partner (duration and nature of relationship, where/ when/ how met partner; if commercial)*
- *Probe around past sexual experiences with this partner. What is the story around the relationship?*

**How is having sex with a “steady partner” different from having sex with others,** such as casual partners, one-night stands, commercial partners, etc
